# Partial Hepatectomy-Induced Upregulation of miR-1907 Accelerates Liver Regeneration by Activation Autophagy

**DOI:** 10.1155/2018/3817057

**Published:** 2018-07-31

**Authors:** Tianfei Lu, Jun Hao, Chuan Shen, Guangxiang Gu, Jianjun Zhang, Ning Xu

**Affiliations:** Department of Liver Surgery, School of Medicine, Shanghai Jiaotong University, Shanghai 200127, China

## Abstract

Liver regeneration after partial hepatectomy (PH) is a highly orchestrated biological process in which synchronized hepatocyte proliferation is induced after massive liver mass loss. Hepatocyte proliferation could be regulated by multiple signals, such as miRNAs and autophagy, but underlying mechanism remains unclear. Here a functional miRNA during liver regeneration was identified and its underlying mechanism was delineated in vitro and in vivo. We found that miR-1907 was highly upregulated during liver regeneration after 2/3 PH at various timepoints. The level of miR-1907 was also increased in normal liver cell line treated with HGF at different concentrations. Functionally, miR-1907 enhanced hepatocyte proliferation in vitro and in vivo, and the liver/body weight ratio in miR-1907-overexpressed mice was significantly higher in comparison to the control mice after 2/3 PH. Forced expression of miR-1907 promoted autophagy activation of hepatocyte. Importantly, autophagy inhibition significantly attenuated miR-1907-induced hepatocyte proliferation and the liver/body weight ratio. Finally, GSK3*β*, a suppressor of autophagy signaling, was identified as the direct target gene of miR-1907. Taken together, miR-1907 accelerates hepatocyte proliferation during liver regeneration by activating autophagy; thus pharmacological intervention regulating miR-1907/autophagy axis may be therapeutically beneficial in liver transplantation and liver failure by inducing liver regeneration.

## 1. Introduction

Liver diseases are a major medical problem for health care systems worldwide [1, 2]. Alcoholism, persistent viral infection, and liver metabolic disorders are the fundamental reason for growing incidence of liver cancer and other liver diseases associated with high mortality [3, 4]. Currently, surgical resection and liver transplantation remain the best option for potential cure and long-term survival in patients with liver cancer and liver failure, respectively [5, 6]. Liver regeneration has great significance because these therapeutic strategies for the surgical treatment of liver diseases depend on the ability of the liver to regenerate physically and functionally [7]. Poor or insufficient liver regeneration may be fatal for these patients [8].

MicroRNAs (miRNAs) are small noncoding RNAs of 18 to 24 nucleotides. They have become firmly established as key regulator of the cells in both normal and pathologic states [9]. Recently, miRNAs have increasingly been reported to regulate the biological process of liver regeneration [10]. Aberrantly expressed miRNAs were identified during liver regeneration. For example, miR-21, miR-23b, miR-122, miR-203, and miR-221 contribute to hepatocyte proliferation, whereas miR-26a, miR-3, miR-34a, miR-150, miR-127, and miR-378 were shown to inhibit hepatocyte proliferation [10]. However, the underlying mechanisms of miRNAs in regulation of liver regeneration remain largely unclear.

Autophagy is a lysosome-mediated intracellular catabolic process by which cells remove their damaged organelles for the maintenance of intracellular homeostasis [11]. Thus autophagy functions as a survival mechanism during cellular stress and contributes to hepatocyte proliferation following PH. Emerging studies reported that autophagy is involved in regulation of liver regeneration [12, 13]. Toshima et al. demonstrated that autophagy plays a crucial role in liver regeneration and in the preservation of cellular quality by preventing hepatocytes from becoming fully senescent and hypertrophic [13]. Lin et al. showed that autophagy is activated in the early stages of liver regeneration following PH* in vivo* [12]. Autophagy inhibition by specific inhibitor aggravates the hepatic injury associated with PH and inhibited hepatocyte proliferation and liver growth. Modulating autophagy might be an effective method of promoting liver regeneration and ameliorating liver injury following PH [12].

Although both miRNAs and autophagy are involved in liver regeneration, little is known about whether miRNAs regulate hepatocyte proliferation and liver growth by activating autophagy during PH. In the present study, we carried out an expression analysis of miRNAs during liver regeneration in mice after PH and identified its mechanism of regulating liver regeneration. We found that miR-1907 was highly upregulated after 2/3 PH and overexpression of miR-1907 promoted hepatocyte proliferation* in vitro* and* in vivo*. Forced expression of miR-1907 increased autophagy activation. More important, autophagy inhibition attenuated miR-1907-induced hepatocyte proliferation.

## 2. Materials and Methods

### 2.1. 2/3 PH (Partial Hepatectomy)

Wild type C57BL/6 mice (6-8 weeks old) were obtained from the Chinese Academy of Sciences (Shanghai, China) and were maintained in a specific pathogen-free facility. All experimental procedures done with mice were approved by the Institutional Care and Use Committee of Renji Hospital. Under isoflurane anesthesia, 2/3 of the liver was surgically removed as described [13].

### 2.2. Cell Lines

Mouse normal hepatocyte lines (CCL-9.1) and human normal hepatocyte lines (HL7702) were obtained from the China Center for Type Culture Collection (Wuhan, China). HL7702 cells were maintained in RPMI-1640 medium (Hyclone, USA) supplemented with 10% FBS (Hyclone, USA) at 37°C in 5% CO2 environment. CCL-9.1 cells were grown in Dulbecco's Modified Eagle Medium (Gibco BRL) with 10% FBS (Hyclone) and were maintained in an atmosphere of 5% CO2 in a humidified 37°C incubator.

### 2.3. miR-1907 Mimics and Inhibitor

miR-1907 mimics (GAGCAGCAGAGGAUCUGGAGGU) or 2′-O-methyl modified miR-1907 inhibitor (ACCUCCAGAUCCUCUGCUGCUC) was transfected to cells by using Lipofectamine™ 2000 (Invitrogen) according to the manufacturer. The effect of miR-1907 mimics or inhibitor on miR1907 overexpression or knockdown has been verified in our laboratory.

miR-1907 (30mg/kg) was mixed with Invivofectamine (1/1, w/v, Invitrogen) and the Invivofectamine-miR-1907 (100 *μ*l) was injected into the tail vein of mice. In the injection experiments, 2/3 PH was performed 7 days after injections. Liver samples of mice injected with miR-1907 were obtained at the indicated time after surgery. The liver and total body weight were measured, and the remnant and regenerated liver tissues were resected and weighed. The acquired data were expressed as a percentage of the ratio between the remnant liver weight (A) divided by the total body weight (B) times 100 (liver-to-body weight ratio (%) = A/B×100). Liver sections were fixed in 10% buffered formalin and processed for PCNA immunohistochemistry as previously described [14].

### 2.4. Quantitative Real-Time PCR (qPCR)

Total RNA of liver specimen and cells was extracted using TRIzol reagent (Invitrogen, CA, USA), and reverse transcription (RT) of miRNA was carried out using miR-1907-special prime. The specific stem-loop RT primers for miR-1907 were designed as previously described [6]. qPCR was performed using a standard protocol from the SYBR Green PCR kit (Toyobo, Osaka, Japan) on Applied Biosystems 7300 real-time PCR system (Applied Biosystems, CA, USA). U6 were used as references for miRNA.

### 2.5. Western Blot Analysis

Equivalent amounts of protein (100 *μ*g) were separated by SDS-PAGE and transferred onto 0.22 *μ*m PVDF membranes (Bio-Rad, CA, USA). The blots were incubated overnight at 4°C with antibodies (LC3B: 2 *μ*g/ml, ab128025, Abcam, MA, USA; p62: 1 *μ*g/ml, ab56416, Abcam; GSK3*β*: 1:1000, ab65740, Abcam; *β*-actin: 1:2000, ab1616, Abcam) and then incubated with a goat anti-rabbit/mouse HRP-conjugated secondary antibody (1:4000, Abcam). Protein signals were visualized by Clarity Western ECL reagent (Bio-Rad). The intensity of the selected bands was quantified using ImageJ software.

### 2.6. Luciferase Reporter Assay

pGL3 plasmid encoding a luciferase reporter gene was purchased from Promega (Madison, WI). Recombinant plasmid of pGL3-GSK3*β*-3′-UTR (wildtype) or pGL3-GSK3*β*-3′-UTR-Mutation was constructed in our laboratory. CCL9.1 cells (1-2 × 10^5^ cells/well) were plated in a 24-well plate and cotransfected with 50 nM of miR-1907, 10 ng of either pGL3-GSK3*β*-3′-UTR or pGL3-GSK3*β*-3′-UTR-Mutation, and 1 ng of pRL-TK (Promega) using Lipofectamine 2000. The pRL-TK vector was used as an internal control to correct the differences in both transfection and harvest efficiencies. Cells were collected 48 h after transfection and analyzed using the Dual-Luciferase Reporter Assay System (Promega).

### 2.7. Fluorescence Microscopy Analysis

Autophagy activation was assessed according to the manufacturers' instructions (ENZ-51031-K200, Enzo Life Science, NY, USA). Briefly, cells were seeded into 12-well plates (1 × 10^4^ cells/well). Following treatment with indicated reagent, cells were washed once in PBS and resuspended in 1× Cyto-ID Green autophagy detection reagent and incubated at room temperature for 30 min. Analysis of the stained cells was performed by wide-field fluorescence (Olympus, Tokyo, Japan).

### 2.8. Cell Proliferation Assay

Cell proliferation was assayed with a Cell Counting Kit-8 (CCK-8, Dojindo, Japan). Cells were plated in 24-well plates in triplicate at about 1 × 10^5^ cells per well and cultured in the growth medium. Cells were treated with indicated reagents and then the numbers of cells per well were measured by the absorbance (450 nm) of reduced WST-8 at the indicated time points. EdU immunofluorescence staining was performed with an EdU kit (Roche) as previously described [10]. BrdU enzyme-linked immunosorbent assay (ELISA) was carried out using a 5-Bromo-2′-deoxy-uridine Labeling and Detection Kit II (Roche) as per the manufacturers' instructions.

### 2.9. Statistics

All data are presented as mean ± standard deviation (SD) from at least three separate experiments. The differences between groups were analyzed using Student's *t*-test. Differences were deemed statistically significant at* p* < 0.05.

## 3. Results

### 3.1. miR-1907 Is Highly Induced during Liver Regeneration

**Figure 1:**
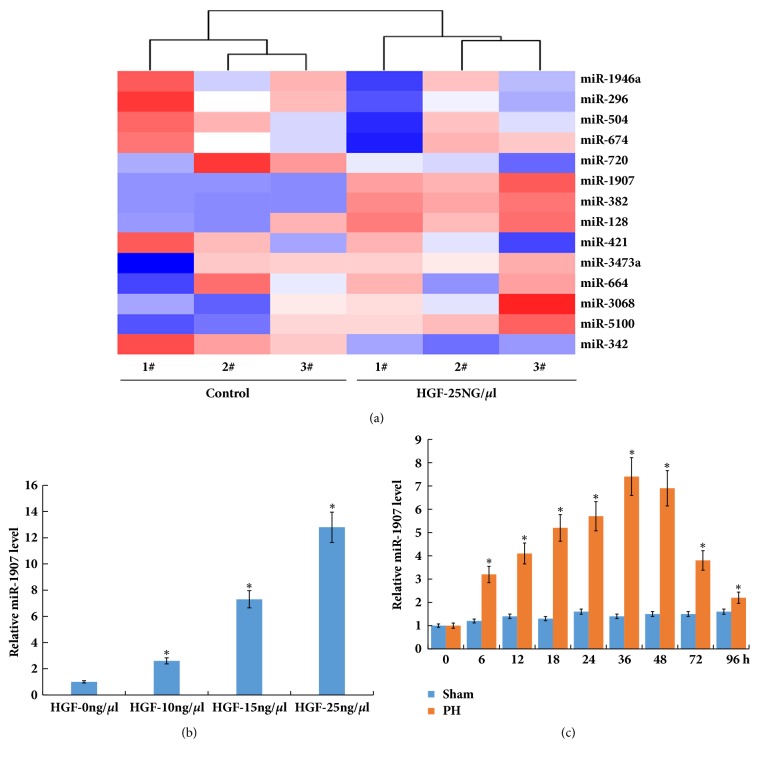
**miR-1907 expression is upregulated after HGF treatment and 2/3 PH**. (a) Heatmap showed the aberrantly expressed miRNAs in CCL-9.1 cells following HGF (25ng/*μ*l) treatment compared to control. (b) Primary mouse hepatocytes were isolated and treated with HGF, and qPCR analysis of miR-1907 expression in primary mouse hepatocytes was carried out with different concentration. (c) qPCR analysis of miR-1907 expression in liver tissues at different timepoints after 2/3 PH. *∗p*<0.05.

miRNAs play a vital role in regulation of various cellular activities which may provide distinct insights into high regenerative ability of the liver. Recently, a miRNA microarray analysis was carried out to evaluate miRNA expression profiles in mouse liver after 2/3 PH [10]. 14 differentially expressed miRNAs (miR-1946a, miR-296, miR-504, miR-128, miR-674, miR-421, miR-3473a, miR-1907, miR-382 miR-3068, miR-664, miR-342-5p, miR-5100, and miR-720) were identified in the miRNA microarray analysis. Due to the important role of hepatocyte growth factor (HGF) in liver regeneration [15, 16], these miRNAs expression levels in CCL-9.1 cells (a normal mouse liver cell line) were assayed after HGF (25 ng/uL) treatment. Among the 14 miRNAs, the expression levels of miR-382 and miR-1907 were significantly increased by HGF treatment (Figure 1(a)). Here miR-1907 was selected for further analysis because the role of miR-382 in regulation of hepatocyte proliferation has been verified [10].

Primary mouse hepatocytes were isolated and then treated with HGF. As shown in Figure 1(b), miR-1907 level was upregulated after HGF treatment in a dose-dependent manner in primary hepatocytes. We further analyzed miR-1907 level in purified hepatocytes from the mouse liver samples at various timepoints.  Figure 1(c) showed that miR-1907 level was markedly upregulated at various timepoints after 2/3 PH. The 2/3 PH in mice resulted in an increase of miR-1907 that was detectable at 6 h, peaked between 36 and 48 h, and returned to almost normal levels by 96 h. The timing of the miR-1907 surge indicated that it might be involved in liver regeneration.

### 3.2. miR-1907 Promotes Hepatocyte Proliferation* In Vitro *and* In Vivo*

**Figure 2:**
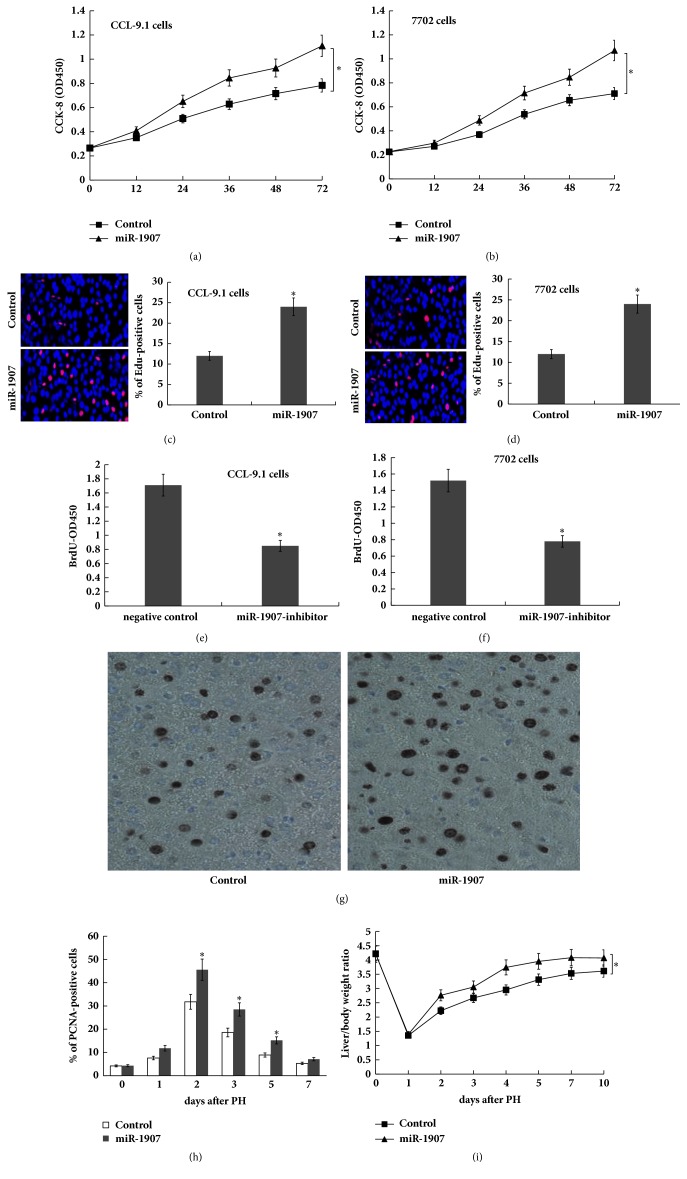
**miR-1907 promotes hepatocyte proliferation* in vitro* and* in vivo***. miR-1907 were overexpressed in CCL-9.1 (a) or in HL7702 (b) using miR-1907 mimics, and cell proliferation was assessed using the CCK-8 assay. miR-1907 were overexpressed in CCL-9.1 (c) or in HL7702 (d), and cell proliferation was assessed using EdU immunofluorescence staining. miR-1907 were overexpressed (e) or inhibited (f) in CCL-9.1, and cell proliferation was assessed using BrdU ELISA. (g) Immunohistochemistry analysis for PCNA showed the differences in hepatocyte proliferation between mice injected with miR-1907 and control. Original magnification ×200. Quantification of PCNA-positive cells at different timepoints after 2/3 PH was showed in (h). n=5. (i) Mice were injected with miR-1907 or control through the tail vein before and after 2/3 PH. The liver mass to body weight ratio was then calculated at different timepoint. *∗p*<0.05.

To investigate the role of miR-1907 in hepatocyte proliferation, Cell Counting Kit-8 (CCK-8) assays, EdU immunofluorescence, and bromodeoxyuridine (BrdU) enzyme-linked immunosorbent assay (ELISA) were carried out in normal mouse/human liver cell line (CCL-9.1 cells and HL-7702 cells) treated with miR-1907 mimics or miR-1907 inhibitor. The results from CCK-8 assays (Figures 2(a) and 2(b)) and EdU assays (Figures 2(c) and 2(d)) showed that hepatocyte proliferation was significantly increased by the overexpression of miR-1907 in CCL-9.1 and HL-7702 cells. The BrdU ELISA assays indicated that hepatocyte proliferation was reduced by the inhibition of miR-1907 (Figures 2(e) and 2(f)). Next,* in vivo* hepatocyte proliferation was analyzed by immunohistochemistry for proliferating cell nuclear antigens (PCNAs). Figures 2(g) and 2(h) showed that miR-1907-treated mice exhibited higher numbers of PCNA-positive nuclei in hepatocytes compared to controls after 2/3 PH for 36 h, which coincides with the peak of miR-1907 expression (Figure 1(c)). Furthermore, we assayed the role of miR-1907 in regulation of liver regeneration* in vivo* through tail vein injection of miR-1907. As shown in Figure 2(i), the liver/body weight ratio in the miR-1907-treated mice was significantly higher compared to the control mice after 2/3 PH. These data suggest that forced expression of miR-1907 contributes to hepatocyte proliferation* in vitro *and* in vivo*.

### 3.3. miR-1907 Activates Hepatocyte Autophagy

**Figure 3:**
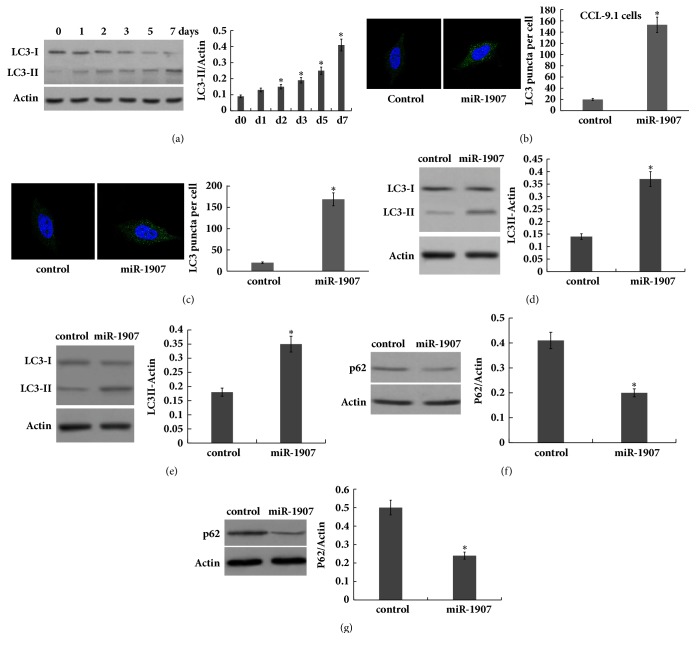
**miR-1907 increases the activation of hepatocyte autophagy**. (a) Western blot analysis of LC-3B expression in liver tissues at different timepoints after 2/3 PH. CCL-9.1 cells (b) or HL7702 (c) cells were treated with miR-1907, and the autophagosome formation was visualized by assaying activated green puncta. Punctate staining is indicative for the redistribution of LC3 to autophagosomes. The average number of green puncta per cell with standard deviation for each group is presented. Scale bar, 50 *μ*m. CCL-9.1 cells (d) or HL-7702 (e) cells were treated with miR-1907 and then western blot analysis of LC-3B protein expression to evaluate autophagy. CCL-9.1 cells (f) or HL7702 (g) cells were treated with miR-1907 and then western blot analysis of p62 protein level to evaluate autophagy. *∗p* < 0.05.

As a survival mechanism during cellular stress, autophagy might play important role in regulating hepatocyte proliferation and liver regeneration after PH. To verify that we first assayed the activation of autophagy in hepatocyte after 2/3 PH. As shown in Figure 3(a), LC3-II expression was gradually increased, indicating that autophagy was gradually activated after 2/3 PH.

Then hepatocytes were treated with miR-1907* in vitro* and respective activation of autophagy was analyzed. Figures 3(b) and 3(c) showed that, following miR-1907 treatment, there was an increase of green puncta representing autophagic vacuoles and an accumulation of LC3-II in CCL-9.1 and HL-7702 cells, indicating that autophagy was activated. The ratio of LC3-II to *β*-actin has been shown to be a reliable indicator of autophagy [17]. Figures 3(d) and 3(e) further showed that miR-1907 treatment resulted in an increase in the ratio of LC3-II/*β*-actin. The ubiquitin-binding protein p62/SQSTM1 is an autophagy substrate, which upon direct binding to LC3 incorporates into autophagosomes and is efficiently degraded by autophagy [18]. The autophagy flux was further verified by assessing the decrease of p62 protein level, a well-established autophagy substrate (Figures 3(f) and 3(g)).

### 3.4. miR-1907 Accelerates Liver Regeneration by Targeting GSK3*β* and Activating Autophagy

**Figure 4:**
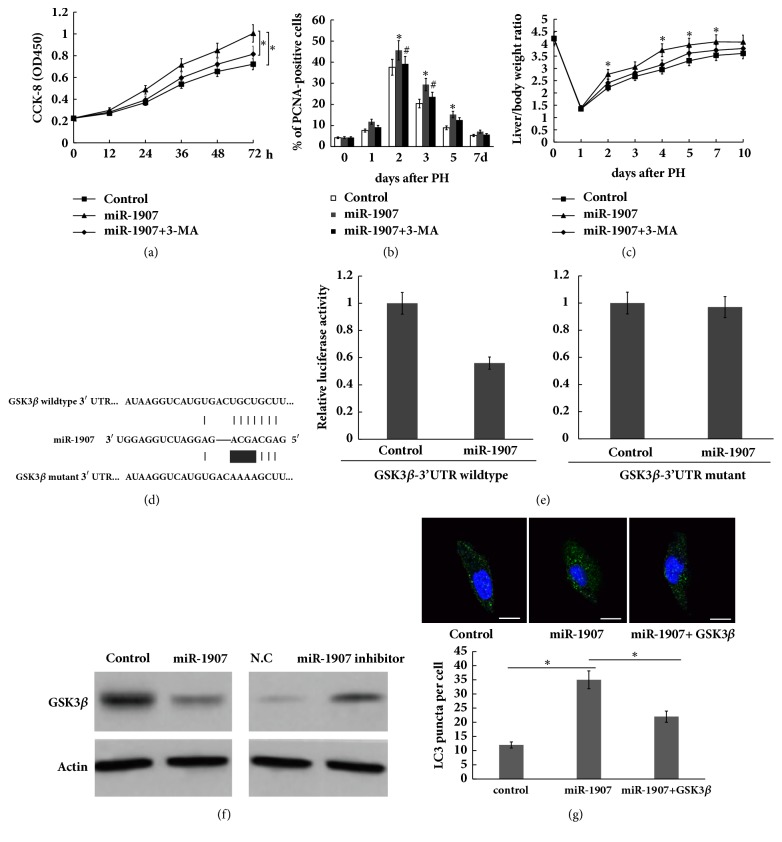
**miR-1907 activates hepatocyte autophagy by inhibiting GSK3** **β**. CCL-9.1 cell proliferation was assayed after miR-1907 treatment with or without 3-MA (1 *μ*M). (b) Quantification of PCNA-positive cells in mice liver injected with miR-1907 with or without 3-MA (2mg/kg) at different timepoints before and after 2/3 PH. (c) Mice were injected with miR-1907 with or without 3-MA before and after 2/3 PH. The liver mass to body weight ratio was then calculated at different timepoint. (d) Schematic representation of the miR-1907 site in GSK3*β* 3′-UTR. (e) The 3′UTR reporter assay was carried out in CCL-9.1 cells overexpressed with miR-1907. pGL3-GSK3*β*-3′-UTR-WT or pGL3-GSK3*β*-3′-UTR-Mutation was cotransfected with pRL-TK. Luciferase assays were performed 48 h after transfection. Firefly luciferase activity was standardized to Renilla luciferase control. (f) Western blot analysis for endogenous GSK3*β* protein level after miR-1907 overexpression or inhibition in CCL-9.1 cells. (g) CCL-9.1 cells were treated with miR-1907 with or without 3-MA, and the autophagosome formation was visualized by assaying activated green puncta. Scale bar, 50 *μ*m. *∗p* < 0.05.

To investigate the role of miR-1907/autophagy signaling in regulation of liver regeneration after 2/3 PH, miR-1907 was overexpressed and autophagy was inhibited simultaneously with specific inhibitor (3-MA), and hepatocyte proliferation and liver regeneration were assayed* in vitro *and* in vivo*. As shown in Figure 4(a), CCL-9.1 cell proliferation was significantly increased after miR-1907 overexpression, whereas autophagy inhibition by 3-MA inhibited the role of miR-1907 in promoting cell proliferation.* In vivo* hepatocyte proliferation was analyzed by immunohistochemistry for PCNAs.  Figure 4(b) showed that miR-1907-treated mice exhibited significantly higher numbers of PCNA-positive nuclei in hepatocytes compared to controls after 2/3 PH for 48 h and 72 h, whereas autophagy inhibition partially attenuated the role of miR-1907 in promoting hepatocyte proliferation. Furthermore, 3-MA treatment suppressed the role of miR-1907 in promoting liver regeneration (Figure 4(c)).

miRNAs inhibits gene expression and then exerts its biological function by binding to specific sites within the 3′-UTR. Here we screened the target genes of miR-1907 using Targetscan 7.1 (http://www.targetscan.org/vert_71/) and selecting autophagy-related genes.* GSK3β* was showed as a potential target gene because GSK3*β* has a binding site of miR-1907 (Figure 4(d)), and previous studies demonstrated that GSK3*β* inhibition contributes to autophagy activation [14, 19–21]. To investigate whether miR-1907 directly binds the 3′-UTR of* GSK3β* and inhibits its expression, we constructed luciferase reporter vector containing 3′-UTR of* GSK3β* (*GSK3β*-3′UTR-LUC). The reporter assay showed that miR-1907 was able to significantly repress luciferase expression of* GSK3β*-3′UTR-LUC, whereas mutation of 4 nucleotides in* GSK3β-*3′-UTR led to complete abrogation of the suppressive effect (Figure 4(e)). Forced expression of miR-1907 repressed GSK3*β* protein level, whereas miR-1907 inhibitor treatment resulted in an increase of GSK3*β* protein level in CCL9.1 cells (Figure 4(f)). Functionally, GSK3*β* overexpression attenuated miR-1907-induced autophagy activation in CCL9.1 cells (Figure 4(g)). These results demonstrate that PH-induced upregulation of miR-1907 accelerates hepatocyte proliferation and liver regeneration by inhibiting GSK3*β* and activation autophagy.

## 4. Discussion

In the study, we investigated the role of miR-1907 in regulation of hepatocyte proliferation and liver regeneration after 2/3 PH. The current data demonstrate that (i) miR-1907 was highly upregulated during liver regeneration after 2/3 PH, (ii) miR-1907 promoted hepatocyte proliferation and increased the liver/body weight ratio, (iii) forced expression of miR-1907 promoted autophagy activation, and (iv) autophagy inhibition significantly attenuated miR-1907-induced hepatocyte proliferation and liver regeneration. These results reveal a function of miR-1907/autophagy axis in regulating hepatocyte proliferation and thus pharmacological intervention regulating miR-1907/autophagy may be therapeutically beneficial in liver transplantation and liver failure by inducing liver regeneration.

miRNAs are class of small noncoding RNAs of 19-24 nucleotides, which negatively regulate protein-coding genes expression and have recently emerged as potent regulators of cell development, differentiation, and proliferation. In particular, the role of miRNAs in tumor biology has been widely investigated [22, 23]. miRNA expression profiles differ between malignant tissues and adjacent benign tissues [24]. Lots of studies showed the association of miRNAs with hepatocellular carcinoma (HCC). For example, miR-221, miR-222, miR-17-92, miR-224, and miR-21 are frequently increased in HCC tumors, whereas miR-122, miR-123, let-7, miR-200, miR-29, miR-199a, and miR-199b are downregulated [25–27]. Detectable in hepatocellular carcinoma tissue, serum, and urine, miRNAs thus provide a minimally invasive way to monitor response to therapy and establish prognosis [24]. Additionally, the roles of miRNA in regulation of autoimmune liver diseases, drug-induced liver injury, alcoholic liver disease, and liver fibrosis have been widely studied [24].

Recently the function of miRNAs in liver regeneration has been gradually revealed. Bei et al. identified a marked upregulation of miR-382 in the mouse liver after PH [10]. miR-382 overexpression accelerates hepatocyte proliferation and G1 to S phase transition of the cell cycle via targeting PTEN-Akt axis. Except miR-382, several other miRNAs have been identified as abnormally expressed after PH. To further identify functional miRNAs during liver regeneration, the expression levels of these miRNAs which dysregulated after PH were assayed after HGF treatment owing to the important role of HGF in liver regeneration [15, 16]. The expression levels of miR-382 and miR-1907 were significantly increased by HGF treatment among these miRNAs. Functionally, overexpression of miR-1907 significantly increased hepatocyte proliferation, whereas inhibition of miR-1907 reduced hepatocyte proliferation.* In vivo* hepatocyte proliferation was analyzed by immunohistochemistry for PCNAs. Current data showed that miR-1907-treated mice exhibited higher numbers of PCNA-positive nuclei in hepatocytes compared to controls after 2/3 PH. Furthermore, the liver/body weight ratio in the miR-1907-treated mice was significantly higher compared to the control mice after 2/3 PH at various timepoints.

Autophagy might play important role in regulating hepatocyte proliferation and liver regeneration after PH because autophagy functions as a cellular survival mechanism during cellular stress. In the study we demonstrated that autophagy was gradually activated following miR-1907 treatment, whereas miR-1907 inhibition suppressed autophagy activation of hepatocyte. To investigate whether miR-1907 promotes hepatocyte proliferation and liver regeneration by regulating autophagy, miR-1907 was overexpressed and autophagy was inhibited simultaneously with 3-MA, and hepatocyte proliferation and liver regeneration were assayed* in vitro* and* in vivo*. As expected, autophagy inhibition by 3-MA inhibited the role of miR-1907 in promoting cell proliferation* in vitro* and* in vivo*. More importantly, 3-MA treatment suppressed the role of miR-1907 in promoting liver regeneration* in vivo*. Finally, GSK3*β*, a suppressor of autophagy signaling, was identified as the direct target gene of miR-1907.** Conclusion**: miR-1907 accelerates hepatocyte proliferation during liver regeneration by activating autophagy; thus pharmacological intervention regulating miR-1907/autophagy axis may be therapeutically beneficial in liver transplantation and liver failure by inducing liver regeneration.

## Data Availability

The data used to support the findings of this study are available from the corresponding author upon request.

## Abbreviations

PH: Partial hepatectomy
miRNAs: MicroRNA.
